# Genome-Wide Identification and Characterization of APETALA2/Ethylene-Responsive Element Binding Factor Superfamily Genes in Soybean Seed Development

**DOI:** 10.3389/fpls.2020.566647

**Published:** 2020-09-04

**Authors:** Wenbo Jiang, Xuejing Zhang, Xuewei Song, Junfeng Yang, Yongzhen Pang

**Affiliations:** ^1^Institute of Animal Science, Chinese Academy of Agricultural Sciences, Beijing, China; ^2^Key Laboratory of Plant Resources and Beijing Botanical Garden, Institute of Botany, Chinese Academy of Sciences, Beijing, China

**Keywords:** *Glycine max*, APETALA2/ethylene-responsive element binding factor, flowering, seed weight, seed size

## Abstract

*Glycine max* is one of the most important grain and oil crops, and improvement of seed yield is one of the major objectives in soybean breeding. The AP2/ERF superfamily members are involved in regulating flower and seed development in many species, and therefore play key roles in seed yield. However, it is still unknown that how many AP2/ERF members were presented in the *G. max* genome and whether these AP2/ERF family members function in flower and seed development in *G. max*. Here, we identified 380 AP2/ERF superfamily genes in the *G. max* genome. Phylogenetic analysis showed that 323 members were grouped into the ERF family, and 49 into the AP2 family. Among the AP2 family, 14 members of the euAP2 lineage showed high identity with their orthologs, and eight member of the ANT lineage were expressed highly in the seeds. Furthermore, seven of them (*GmAP2-1* to *GmAP2-7*) were successfully cloned and over-expressed in *Arabidopsis thaliana*. The transgenic *Arabidopsis* plants over-expressing these *GmAP2* genes flowered earlier relative to the wild type control. The seed length and width, and seed area of these over-expression lines were increased compared with the wild type, and seed weight of over-expression lines of *GmAP2-1*, *GmAP2-4*, *GmAP2-5*, and *GmAP2-6* were greater than those of the wild type. Furthermore, the seed number per silique of the over-expression lines for *GmAP2* genes were not affected except *GmAP2-5*. Collectively, *GmAP2-1*, *GmAP2-4*, and *GmAP2-6* played important roles in regulating seed weight by affecting seed length, width and area, and further controlling seed yield.

## Introduction

Soybean (*Glycine max*) was domesticated in China more than 5,000 years ago, which is one of the most important grain and oil crops. Improvement of seed yield is one of the major objectives in soybean breeding. Soybean is typically a short-day sensitive plant (Fehr and Caviness, 1977; Fehr, 1987), and both the time of flowering and date of seed maturity underlie seed yield (Yuan et al., 2002). Previous studies have demonstrated that the *APETALA2* (*AP2*) gene in *Arabidopsis thaliana* regulates floral development (Jofuku et al., 1994b; Chen et al., 2004; Wollmann et al., 2010; Yant et al., 2010; Dinh et al., 2012; Krogan et al., 2012; Liu et al., 2014; Huang et al., 2017), ovule and seed development, and subsequently mediates seed size and seed weight (Jofuku et al., 1994b; Jofuku et al., 2005; Ohto et al., 2005; Ohto et al., 2009). Besides *AP2* in *Arabidopsis*, its orthologs in other plant species were also found to be able to regulate seed-related phenotypes. For an example, over-expression of *LaAP2L1* from *Larix* led to over 200% greater seed yield in *Arabidopsis*, contributed by enhanced cell proliferation and prolonged growth duration (Li et al., 2013). The *AP*2-like genes in *Petunia* regulates flower and seed development (Maes et al., 2001). Over-expression of the *AfAP2-2* gene from *Aechmea fasciata* reduces seed size and delays flowering in *Arabidopsis* (Lei et al., 2019). All of the above-mentioned *AP2* genes belong to the APETALA2/ethylene-responsive element binding factor (AP2/ERF) superfamily, which suggested that AP2/ERF superfamily members have potential impact on seed yield in soybean as in other plant species.

The AP2/ERF superfamily is a plant-specific transcription factor family containing a large number of members with at least one AP2 domain. This superfamily can be mainly divided into ERF, RAV, and AP2 families based on the numbers of the conserved AP2 domain and other DNA binding domains (Riechmann and Meyerowitz, 1998; Mizoi et al., 2012). The ERF family members consist of two subfamilies (ERF and DREB) with a single AP2 domain. The RAV family members have a single AP2 domain and an additional B3 domain. The AP2 family members contain a double and tandemly repeated AP2 domain (Sakuma et al., 2002; Nakano et al., 2006; Mizoi et al., 2012; Shu et al., 2015).

The ERF family members mainly respond to abiotic or biotic stresses in soybean and other plant species. For example, *GmSGR*, a seed-specific ERF family member in *G. max*, reduced ABA sensitivity and enhanced salt sensitivity in the seeds of the transgenic *Arabidopsis* plants by regulating the expression levels of the *AtEm6* and *AtRD29B* genes (Wang et al., 2008). Over-expression of the *GmERF3* gene increased tolerances to salt, drought, and diseases in transgenic tobacco plants (Zhang et al., 2009). Both *GmERF5* and *GmERF113* enhance resistance to pathogen *Phytophthora sojae* in *G. max* (Dong et al., 2015; Zhao et al., 2017). *GsERF6* from *G. soja* significantly enhanced tolerance to bicarbonate in the transgenic *Arabidopsis* plants (Yu et al., 2016a). *GsERF71* positively regulates alkaline stress tolerance in *Arabidopsis* (Yu et al., 2017). *GsSnRK1* interplays with *GsERF7* to regulate stress resistance in *G. soja* (Feng et al., 2020). These studies on ERF family members showed that ERF proteins mainly involved in abiotic and biotic stresses in soybean. However, a recent study showed that one ERF family member Glyma19g192400 is involved in plant growth regulation that mainly affects plant height, while another ERF member Glyma19g163900 is related to seed weight, which is involved in maintaining seed size, embryo size, seed weight and seed yield (Assefa et al., 2019).

The RAV family members regulate diverse processes, including cold tolerance, dehydration and circadian rhythm (Matias-Hernandez et al., 2014). In *Arabidopsis*, TEMPRANILLO1 (TEM1) and TEM2, two members of the RAV family, were identified as repressors in floral induction (Osnato et al., 2012). In *G. max*, *GmRAV* controls photosynthesis and senescence (Zhao et al., 2008), and *GmRAV1* regulates the regeneration of roots and adventitious buds in both transgenic *Arabidopsis* and soybean plants (Zhang et al., 2019).

The AP2 family proteins are divided into two lineages: the ANT lineage and the euAP2 lineage (Kim et al., 2006). The ANT lineage is supported by a 10-aa insertion in the AP2-R1 domain and a 1-aa insertion in the AP2-R2 domain, relative to all other members of the AP2 family (Kim et al., 2006). The euAP2 lineage contains six members (*AP2*, *TOE1*, *TOE2*, *TOE3*, *SMZ*, and *SNZ*) that are targeted by a major developmental microRNA172 (miR172), which functions as a floral promoter (Park et al., 2002; Aukerman and Sakai, 2003; Schmid et al., 2003; Chen et al., 2004). The *ap2* mutant and the quadruple mutant (*toe1/toe2/smz/snz*) flowered earlier than the wild type, but did not flower as early as the 35S:miR172 lines. The flowering time of the hexuple mutant (*ap2*/*toe1/toe2/toe3/smz/snz*) showed no difference from the 35S:miR172 plants (Yant et al., 2010). These results indicate that all the six members of the euAP2 lineage regulate the flowering time in *Arabidopsis*, and they were all under the control of miR172.

Among the AP2 family members, the *AP2* gene is expressed in all four floral organs including sepal, petal, stamen and carpel, and in the developing ovules, which plays an important role in the control of flower, fruit and seed development (Jofuku et al., 1994b; Ripoll et al., 2011). In addition, the *AP2* gene also functions in stem cell maintenance in shoot apical meristem (Würschum et al., 2006) and the control of floral stem cells (Aukerman and Sakai, 2003; Chen, 2004). The *AP2* gene also mediates seed size by affecting embryo, endosperm, and seed coat development (Jofuku et al., 2005; Ohto et al., 2005; Ohto et al., 2009).

Most advances on the *AP2* genes were carried out in *Arabidopsis*, while only few studies characterized the function of the *AP2* orthologs in other plant species. For example, the double mutant *lip1/lip2* in *Antirrhinum majus* displays homeotic conversion from sepals to leaves, leaving petals intact, which indicated that these two genes have a partially conserved role in floral organ identity with *AtAP2* (Keck et al., 2003). The ortholog of *AtAP2* in *Petunia*, *PhAP2A*, is expressed in a similar pattern as *AtAP2* during flower development. However, the *phap2a* mutant does not display the same phenotypes as the *Arabidopsis ap2* mutant due to the existence of other redundant AP2 members in *Petunia* (Maes et al., 2001). The *AtAP2* ortholog in *Solanum lycopersicum*, *SlAP2a*, is a negative regulator of tomato fruit ripening (Chung et al., 2010). In *G. max*, only one AP2 family member Glyma01g022500 was reported to be associated with shoot related development and it affected internode number, but the molecular mechanism of this gene associated with internode number is still unclear (Assefa et al., 2019). It is still unknown that how many AP2 members were presented in the *G. max* genome, and whether these AP2 family members function in flower or seed development.

In this study, the protein sequences of all putative AP2/ERF superfamily members were analyzed in comparison with those of the *Arabidopsis* orthologs. Among them, seven *AP2* genes (*GmAP2-1* to *GmAP2-7*) were further characterized by ectopic over-expression in *Arabidopsis*. The seed phenotypes including the seed length and width, seed area and size were affected at different levels in these transgenic lines. Our study established an atlas of the AP2/ERF superfamily members in soybean, which will facility in-depth investigation on functions of *AP2* gene members and the utilization in molecular breeding of soybean with improved seed yield.

## Materials and Methods

### Plant Material and Growth Condition

Soybean material used in this study is the cultivar Williams 82. The *ap2-6* mutant of *AP2* gene (At4g36920) and transgenic *Arabidopsis* plants are in the Col-0 background. *Arabidopsis* seeds were chilled at 4°C in the dark for 3 days, and then grown under long-day conditions (16-h light/8-h dark) at 22°C in growth chamber and seeds were harvested when the plant was completely mature. Soybean seeds were grown under long-day conditions (16-h light/8-h dark) at 25°C in growth chamber.

### Phylogenetic Analysis

Multiple sequence alignment was performed by using ClustalX. The neighbor-joining (NJ) method was applied to construct trees using MEGA X software (Kumar et al., 2018). Bootstrapping with 500 replications was performed. The amino acid sequences of AP2 are retrieved from the Arabidopsis database (https://www.arabidopsis.org/) and NCBI database (http://www.ncbi.nlm.nih.gov/). All the amino acid sequences of soybean are retrieved from the soybean database (http://soykb.org/).

### Multiple Sequence Alignment

The multiple sequence alignment was performed by using the software DNAMAN. The accession number are as followed (shown in parenthesis): *Arabidopsis* AtAP2 (AT4G36920); *Glycine max* GmAP2-1 (Glyma01g39520.3), GmAP2-2 (Glyma05g18041.1), GmAP2-3 (Glyma15g04930.1), GmAP2-4 (Glyma13g40470.1), GmAP2-5 (Gm16g00950.2), GmAP2-6 (Glyma08g38190.2), and GmAP2-7 (Glyma15g34770.1).

### Gene Expression Analysis

Total RNAs were isolated from roots, stems, leaves, flowers, and seeds at different development stages: 10, 20, 30, 40, 50, 60, 70, and 80 day after pollination (DAP) of reference cultivar Williams 82, and also isolated from the transgenic lines of each gene in *Arabidopsis*. Semi-quantitative RT-PCR was carried out on Applied Biosystems Veriti Thermal Cycler. The PCR conditions were 94°C for 3 min; 20-30 cycles of 94°C for 20 s, 60°C for 20 s, and 72°C for 20 s; followed by a final extension of 72°C for 10 min. Quantitative RT-PCR analyses were carried out on Applied Biosystems 7500 Real-Time PCR Systems by using the SYBR Green reagent (Takara) according to the manufacturer’s instructions. The *PP2A* gene (*Protein Phosphatase 2A subunit A3*, At1g13320) in *Arabidopsis* and the *SUB3* gene (*Soybean ubiquitin-3*, Glyma20g27950) in soybean were used as the reference genes. The primer sequences used for RT-PCR were listed in **Supplementary Table S2**. Data were calculated from three biological replicates, and each biological replicate was examined in triplicate.

### RNA Isolation, Gene Cloning, and Plasmid Construction

Total RNAs were prepared by using the TRNzol Reagent (TIANGEN) according to the manufacturer’s instructions. First strand cDNA was synthesized using the HiFiScript reverse transcriptase (CWBIO). Fragments of candidate *GmAP2* genes were amplified using the cDNA templates from soybean cultivar Williams 82 seeds with the gene specific primers (**Supplementary Table S1**). PCRs were carried out using Phusin High-Fidelity DNA Polymerase (NEB). Reactions were performed at 98°C for 2 min, and 98°C for 10 s, 56-64°C for 20 s, 72°C for 60 s for 35 cycles, and then 72°C for 7 min. The PCR products were cloned into pENTR entry vector (Invitrogen) and then subcloned into pB7WG2D by the LR reaction to generate the plant over-expression vectors, which were further confirmed by sequencing.

### Generation of the Transgenic *Arabidopsis*

The over-expression vectors constructed as above mentioned were transformed into the *Agrobacterium tumefaciens* strain GV3101, and used for the transformation using the floral-dip method (Clough and Bent, 1998). The transformed plants were grown in the growth chamber, and the seeds were collected and screened on MS medium supplemented with 10 mg/L PPT for 2-3 days at 4°C, and then transformed to a growth chamber at 22°C and a 16/8 h photoperiod for 5-7 days. The seedlings were selected and transferred to sterile soil, and grown at 22°C and a 16/8 h photoperiod. The seeds were harvested from individual T_1_ generation transgenic *Arabidopsis* plants, and DNA was isolated from leaf using CTAB method. Positive transgenic lines were further identified by PCR with gene-specific primers. Then we used the same PCR methods to select T_2_ generation until getting homozygous T_3_ generation transgenic *Arabidopsis* plants.

### Phenotypes of the Transgenic *Arabidopsis* Plants

The wild-type and transgenic *Arabidopsis* plants were grown in the same tray to ensure the same growth condition (16-h light/8-h dark) at 22°C. The seed number was calculated from 36 siliques for each line. The rosette leaf number of flowering and bolting time of transgenic lines were detected with 10 plants for each line.

### Measurement of Seed Weight, Length, Width, and Area

Measurement of seed weight, length, width, and area were performed as previously described (Jiang et al., 2013), with minor modifications. For seed weight, plants were grown concurrently under identical conditions and seeds were harvested when the seeds were completely mature. One thousand seeds per transgenic line were dried at 37°C for 6 days and weighed. Data are presented as means ± SD from at least three independent experiments. For seed length and width, dried seeds were photographed using a Leica M165FC microscope and then measured by ImageJ software. ImageJ software was also used to calculate the seed area using 40 seeds for each line.

### Statistical Analysis

The averages and standard deviations were calculated by using the SigmaPlot 10.0 software (Systat Software, Inc., Chicago, IL, United States). The Student’s t-test was used for *p-*value generation between the wild type and each transgenic *Arabidopsis* lines.

## Results

### Phylogenetic Analysis of the AP2/ERF Superfamily in *G. max*

**Figure 1:**
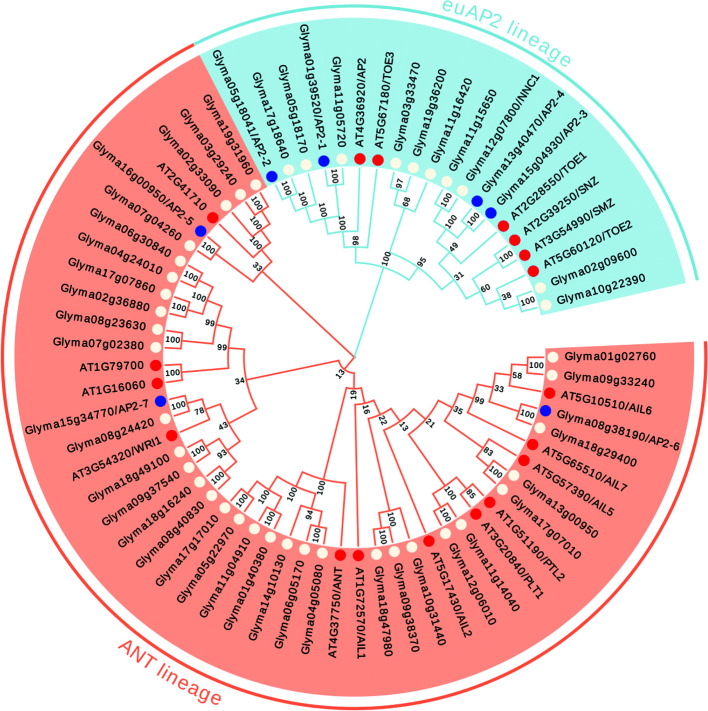
Phylogenetic analysis of the AP2 family members in soybean and *Arabidopsis*. The euAP2 lineage was indicated in blue background, and the ANT lineage was indicated in orange background. The AP2 members in soybean were indicated in white dot with characterized 7 *GmAP2* in blue dot, and the AP2 members in *Arabidopsis* were indicated in red dot. The neighbor-joining (NJ) method was applied to construct trees using MEGA X software. Bootstrapping with 1,000 replications was performed.

To determine the evolutionary relationships of the AP2/ERF superfamily proteins in *G. max*, we obtained the amino acid sequences of 380 AP2/ERF superfamily members from the soybean genome database (http://soykb.org/), and 145 AP2/ERF superfamily protein sequences were also retrieved from The Arabidopsis Information Resource (TAIR) and analyzed in parallel. The phylogenetic tree was constructed based on the alignment of the full-length amino acid sequences of all these 525 proteins (**Figure 1**, **Supplementary Figures S1** and **S2**).

Among these family members, 49 proteins from *G. max* and 18 from *Arabidopsis* were assigned to the cluster of the AP2 family, due to the presence of the tandemly repeated double AP2 domain (**Figure 1**). Three hundred and twenty three proteins from *G. max* and 122 from *Arabidopsis* were grouped into the ERF family, and they all contain a single AP2 domain (**Figure 1**, **Supplementary Figure S1**). Eight proteins from *G. max* and five from *Arabidopsis* were clustered as the RAV family, with the presence of a single AP2 domain together with a B3 domain (**Figure 1**, **Supplementary Figure S2**).

The number of the AP2/ERF superfamily members in *G. max* was 2.62 folds more than that of the *Arabidopsis*. In particular, the numbers of the AP2 and ERF family genes in *G. max* were 2.72 and 2.65 folds more than those in *Arabidopsis*, respectively. Compared with *Arabidopsis*, these two family members of AP2/ERF superfamily proteins in *G. max* appeared to increase proportionally during evolution.

### Screening of the Candidate *AP2* Genes in *G. max*

Previous studies showed that the *AP2* gene (At4g36920) in *Arabidopsis* regulated flower and seed development (Jofuku et al., 1994b; Jofuku et al., 2005; Ohto et al., 2005; Ohto et al., 2009). In addition, the other five genes (*TOE1*, *TOE2*, *TOE3*, *SMZ* and *SNZ*) in the same euAP2 lineage with *AP2*, also regulate flower development in *Arabidopsis* (Yant et al., 2010). Based on these studies and the phylogenetic relationship, we found that 14 genes from *G. max* (Glyma01g39520, Glyma02g09600, Glyma03g33470, Glyma05g18041, Glyma05g18170, Glyma10g22390, Glyma11g05720, Glyma11g15650, Glyma11g16420, Glyma12g07800, Glyma13g40470, Glyma15g04930, Glyma17g18640 and Glyma19g36200) were clustered together with the six genes (*AP2*, *TOE1*, *TOE2*, *TOE3*, *SMZ*, and *SNZ*) of the euAP2 lineage from *Arabidopsis* in the same clade (**Figure 1**). Therefore, these 14 genes are most likely the AP2 orthologs in *G. max* for the regulation of flower and seed development.

To further identify additional members in the AP2 family that are potentially involved in seed development in *G. max*, we also retrieved the expression profiles of all the ANT lineage genes from the online database (https://soybase.org/soyseq/), and found that eight genes (Glyma01g02760, Glyma07g04260, Glyma08g24420, Glyma08g38190, Glyma13g00950, Glyma15g34770, Glyma16g00950, and Glyma18g29400) were expressed significantly higher in seeds than in other tissues (**Table 1**). The typical expression profile indicated that these eight genes of the ANT lineage may be also involved in regulating seed development in *G. max*.

**Table 1:** The relative transcript level of AP2 family genes in different tissues of soybean.

Gene locus	Gene name	Lineage	YL	R	N	F	P	PS10	PS14	S10	S14	S21	S25	S28	S35	S42
**Glyma01g39520**	GmAP2-1	euAP2 lineage	**169**	**200**	**19**	**116**	**135**	**130**	**85**	**42**	**35**	**13**	**45**	**39**	**45**	**22**
**Glyma05g18041**	GmAP2-2		no	no	no	no	no	no	no	no	no	no	no	no	no	no
**Glyma15g04930**	GmAP2-3		**24**	**289**	**37**	**23**	**0**	**1**	**1**	**2**	**2**	**1**	**0**	**1**	**1**	**1**
**Glyma13g40470**	GmAP2-4		**55**	**171**	**79**	**12**	**3**	**0**	**2**	**5**	**6**	**5**	**12**	**14**	**13**	**3**
**Glyma12g07800**	GmNNC1		**184**	**50**	**5**	**43**	**29**	**38**	**30**	**4**	**5**	**12**	**18**	**6**	**12**	**5**
Glyma02g09600			47	64	6	127	15	13	26	3	0	0	2	1	4	0
Glyma03g33470			25	104	24	55	80	53	56	6	25	15	10	8	10	4
Glyma05g18170			117	81	26	137	128	89	43	42	51	27	37	23	24	11
Glyma10g22390			13	8	0	8	2	3	6	1	2	0	0	0	0	0
Glyma11g05720			169	130	7	183	246	204	117	90	97	39	72	59	55	28
Glyma11g15650			243	114	16	18	10	6	5	0	4	2	3	3	3	2
Glyma11g16420			0	0	1	0	0	0	0	0	0	0	0	0	0	0
Glyma17g18640			110	106	96	235	166	137	82	132	90	27	37	17	54	19
Glyma19g36200			9	197	83	87	15	17	20	12	32	34	38	20	36	33
**Glyma16g00950**	GmAP2-5	ANT lineage	**2**	**0**	**0**	**14**	**4**	**3**	**0**	**18**	**38**	**29**	**76**	**51**	**156**	**88**
**Glyma08g38190**	GmAP2-6		**24**	**1**	**0**	**4**	**10**	**5**	**1**	**34**	**72**	**16**	**52**	**43**	**60**	**31**
**Glyma15g34770**	GmAP2-7		**11**	**30**	**21**	**1**	**1**	**1**	**0**	**14**	**42**	**26**	**108**	**60**	**125**	**27**
**Glyma01g02760**			**21**	**43**	**6**	**2**	**2**	**4**	**3**	**30**	**41**	**26**	**70**	**42**	**72**	**24**
Glyma01g40380			7	16	0	9	12	19	4	3	0	0	1	1	5	0
Glyma02g33090			37	14	10	20	37	24	7	11	26	21	22	10	12	3
Glyma02g36880			0	6	2	0	0	0	0	0	0	0	0	0	0	0
Glyma03g29240			32	4	9	16	20	25	10	17	29	13	21	18	38	35
Glyma04g05080			239	55	9	83	59	48	12	8	13	8	9	3	10	0
Glyma04g24010			0	0	0	0	0	0	0	0	0	0	0	0	0	0
Glyma05g22970			179	39	0	42	58	44	12	43	69	33	55	19	29	17
Glyma06g05170			108	31	5	47	52	50	3	22	21	8	11	3	2	0
Glyma06g30840			0	0	0	0	0	0	0	0	0	0	0	0	0	0
Glyma07g02380			20	11	2	26	13	31	20	0	1	0	0	0	0	0
**Glyma07g04260**			**7**	**0**	**0**	**54**	**41**	**24**	**4**	**9**	**53**	**48**	**167**	**116**	**229**	**115**
Glyma08g23630			18	18	52	10	13	17	9	1	1	0	0	0	0	0
**Glyma08g24420**			**3**	**24**	**6**	**0**	**1**	**2**	**0**	**5**	**34**	**44**	**112**	**63**	**133**	**37**
Glyma08g40830			4	0	0	0	1	1	0	1	0	1	8	2	12	3
Glyma09g33240			35	74	113	4	11	6	2	17	79	60	106	66	95	48
Glyma09g37540			0	0	7	0	0	0	0	1	0	0	0	0	0	0
Glyma09g38370			0	31	0	2	0	1	2	0	9	9	6	6	15	7
Glyma10g31440			0	0	0	0	0	0	0	1	1	0	1	0	0	0
Glyma11g04910			3	15	0	9	3	3	2	1	0	1	0	0	0	0
Glyma11g14040			0	19	0	1	0	0	0	3	8	5	7	1	5	5
Glyma12g06010			0	29	0	0	0	1	0	4	1	1	3	1	3	1
**Glyma13g00950**			**74**	**51**	**7**	**50**	**36**	**31**	**14**	**22**	**46**	**36**	**180**	**115**	**199**	**125**
Glyma14g10130			33	21	35	10	9	8	0	9	19	8	11	1	11	0
Glyma17g07010			50	88	8	54	71	60	25	27	44	31	81	34	61	43
Glyma17g07860			0	7	5	0	0	0	0	0	0	0	0	0	0	0
Glyma17g17010			167	28	8	41	39	17	4	138	135	46	82	49	80	44
Glyma18g16240			6	0	0	1	0	0	0	0	0	1	1	2	7	1
**Glyma18g29400**			**6**	**1**	**4**	**3**	**9**	**4**	**5**	**28**	**42**	**40**	**89**	**102**	**147**	**83**
Glyma18g47980			0	20	0	3	1	2	0	3	6	6	20	7	19	12
Glyma18g49100			0	0	158	0	0	0	0	0	0	0	0	0	1	0
Glyma19g31960			47	17	5	35	19	25	7	3	7	1	20	14	19	11

Data were retrieved from https://soybase.org/soyseq/.

YL, young leave; R, roots; N, nodule; F, flower; P, one cm pod; PS10, pod shell at 10 days after pollination; PS14, pod shell at 14 days after pollination; S10, seeds 10 days after pollination; S14, seeds 14 days after pollination; S21, seeds 21 days after pollination; S25, seeds 25 days after pollination; S28, seeds 28 days after pollination; S35, seeds 35 days after pollination; S42, seeds 42 days after pollination.

We therefore initially amplified all the above mentioned 22 genes by RT-PCR, but only eight of them were successfully cloned and used for further studies. These eight genes (Glyma12g07800, Glyma01g39520, Glyma05g18041, Glyma15g04930, Glyma13g40470, Glyma16g00950, Glyma08g38190, and Glyma15g34770) were further designated in order as *GmNNC1*, *GmAP2-1*, *GmAP2-2*, *GmAP2-3*, *GmAP2-4*, *GmAP2-5*, *GmAP2-6*, and *GmAP2-7*, respectively. Among them, *GmNNC1* were recently found to negatively regulate nodule number by another group (Wang et al., 2014), which will not be further pursued in this study. Therefore, these seven *AP2* genes (*GmAP2-1* to *GmAP2-7*) from *G. max* were further characterized in this study.

### Sequence Analyses of the *GmAP2* Genes

**Figure 2:**
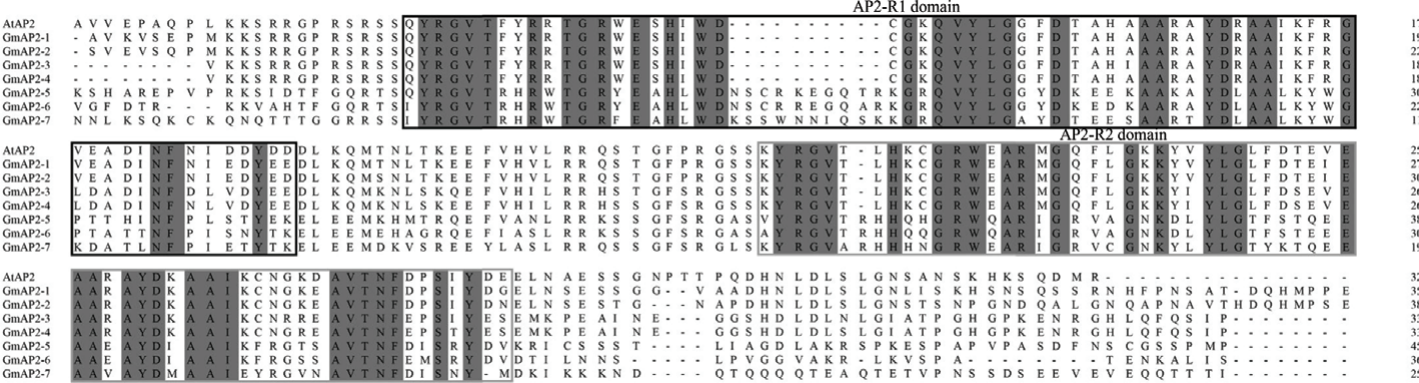
Sequence alignment of the candidate AP2 proteins in soybean with those from *Arabidopsis*. The alignment was performed by using software DNAMAN. The two AP2 domains were boxed, with black color for AP2-R1 domain and grey color for AP2-R2 domain. All the sequences for soybean used in the figure are retrieved from the Soybean Database (http://soykb.org/) and AtAP2 are retrieved from the Arabidopsis Database (https://www.arabidopsis.org/). The accession number are as followed (shown in parenthesis): *Arabidopsis thaliana*: AtAP2 (At4g36920); *Glycine max*: GmAP2-1 (Glyma01g39520.3), GmAP2-2 (Glyma05g18041.1), GmAP2-3(Glyma15g04930.1), GmAP2-4 (Glyma13g40470.1), GmAP2-5 (Gm16g00950.2), GmAP2-6 (Glyma08g38190.2), and GmAP2-7 (Glyma15g34770.1).

To further analyze the seven candidate *GmAP2* genes, their amino acid sequences were aligned with *AP2* gene of *Arabidopsis*. These *GmAP2* proteins contained two AP2 domains (**Figure 2**, **Supplementary Figure S3**), which supported the fact that they belonged to the AP2 family. The deduced GmAP2-1, GmAP2-2, GmAP2-3, GmAP2-4 proteins showed 60.5%, 77.01%, 79.33%, and 57.19% identity with AtAP2 at amino acid level, respectively, and they were clustered with AtAP2 in the phylogenetic tree **(****Figure 1**, **Supplementary Table S2****)**. The deduced GmAP2-5, GmAP2-6, and GmAP2-7 proteins showed relatively low identity with AtAP2 (54.44%, 53.85%, and 52.33%, respectively, **Supplementary Table S1**). Meanwhile, the identity among GmAP2-1, GmAP2-2 and GmAP2-3 is more than 73% at amino acid level, more than 90% between GmAP2-3 and GmAP2-4, and more than 70% among GmAP2-5, GmAP2-6, and GmAP2-7 (**Supplementary Table S1**). It was obvious that four proteins (GmAP2-1, GmAP2-2, GmAP2-3, and GmAP2-4) shared higher sequence identity with AtAP2 than the other three GmAP2 proteins (GmAP2-5, GmAP2-6, and GmAP2-7).

Their AP2 domain regions (two AP2 domains and the linker sequence between them) were also aligned, and the corresponding sequence of GmAP2-1, GmAP2-2, GmAP2-3, and GmAP2-4 showed 96.77%, 96.77%, 86.45%, and 87.74% identity with AtAP2, respectively. The sequence identity in the AP2 domain regions was much higher than in the other regions of these four proteins. However, the sequence identity of the AP2 domain regions in GmAP2-5, GmAP2-6, and GmAP2-7, respectively, were lower as aligned with their full-length amino acid sequence.

### Expression Profiles of the Candidate *GmAP2* Genes

**Figure 3:**
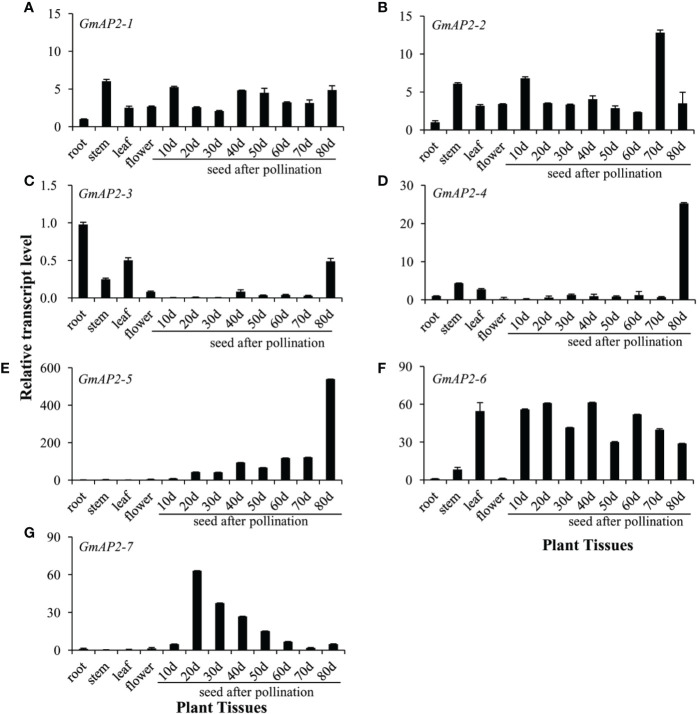
Expression profiles of the candidate AP2 genes in different tissues of *G. max*. Total RNAs were isolated from roots, stems, leafs and flowers, 10 days seeds after pollination (10 days), 20, 30, 40, 50, 60, 70, and 80 days. The relative transcript level in roots for each gene was set as a value of 1.0. The *SUB3* gene (*Soybean ubiquitin-3*, Glyma20g27950) in *G. max* was used as the reference gene. **(A–G)** The relative transcript level of GmAP2-1 **(A)**, GmAP2-2 **(B)**, GmAP2-3 **(C)**, GmAP2-4 **(D)**, GmAP2-5 **(E)**, GmAP2-6 **(F)**, and GmAP2-7 **(G)**, respectively.

In order to further analyze the expression pattern of these candidate *GmAP2* genes, RNAs were extracted from roots, stems, leaves, flowers, and seeds at different developing stages (10, 20, 30, 40, 50, 60, 70, and 80 days after pollination-DAP). The relative expression levels of these eight *GmAP2* genes in different tissues were detected by real time quantitative PCR. The transcript levels of *GmAP2-1* and *GmAP2-2* were relatively stable in all tested tissues (**Figures 3A, B****)**. *GmAP2-3* was expressed higher in roots, leaves and in 80-DAP seeds than in other tissues (**Figure 3C**). The transcript levels of *GmAP2-4* were much higher in 80-DAP seeds than in other tissues (**Figure 3D**). The transcript levels of *GmAP2-5* were relatively low in roots, stems, leaves and flowers, but its expression gradually increased with seed maturity. In particular, the transcript level of *GmAP2-5* reached the highest level in 80-DAP seeds (**Figure 3E**). The transcript levels of *GmAP2-6* were significantly higher in leaves and seeds than in roots, stems or flowers (**Figure 3F**). *GmAP2-7* was mainly expressed in seeds, and its transcripts reached the highest level in 20-DAP seeds, and then gradually decreased afterwards (**Figure 3G**).

Collectively, these results suggested that *GmAP2-1* and *GmAP2-2* might function in different tissues of *G. max*; *GmAP2-3* and *GmAP2-4* might play a major role during late seed developmental stage; *GmAP2-5* might be primarily involved in seed development, especially late stage. By contrast, *GmAP2-7* might function in early seed developmental stage and *GmAP2-6* might play a key role in both leaves and seed development.

### Over-Expression of Seven *GmAP2* Genes in *Arabidopsis*

To functionally characterize these *GmAP2* genes, we individually transformed them into the wild type *Arabidopsis* (Columbia-0). More than 70 over-expression lines for every gene were obtained, and the transgenic plants with a 3:1 (resistant:sensitive) segregation ratio based on the Bar resistance were preselected. The T_3_ homozygous transgenic lines were verified by RT-PCR with gene-specific primers (**Supplementary Figure S4**), and at least six homozygous over-expression lines were further analyzed for each transgene.

**Figure 4:**
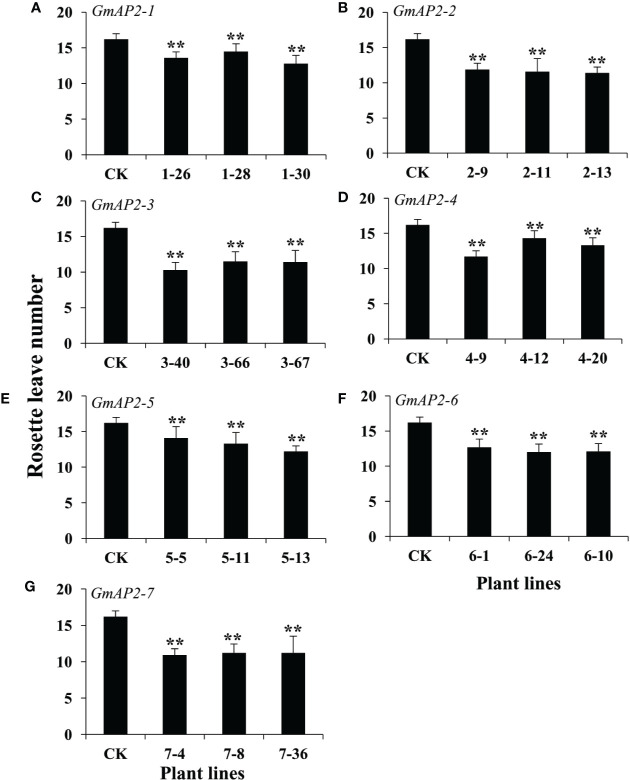
Rosette leave numbers of the transgenic lines over-expressing seven individual *GmAP2* genes in *Arabidopsis*. The number of rosette leaves was calculated at the beginning of bolting to evaluate the flowering time of the transgenic lines. Ten plants for each line were selected to count the number of the rosette leaves. CK indicates the wild type *Arabidopsis*. **(A–G)** The over-expression lines for *GmAP2-1* **(A)**, *GmAP2-2* **(B)**, *GmAP2-3* **(C)**, *GmAP2-4* **(D)**, *GmAP2-5* **(E)**, *GmAP2-6* **(F)**, *GmAP2-7* **(G)**. Data are presented as mean ± SD, Student’s t test (n = 10, **P < 0.01).

Due to their potential roles in the regulation of flowering time, we evaluate the flower time phenotype of these transgenic lines by counting the number of rosette leaves at the beginning of bolting. The number of rosette leaves in the transgenic lines over-expressing *GmAP2-1*, *GmAP2-2*, *GmAP2-3*, *GmAP2-4*, *GmAP2-5*, *GmAP2-6*, and *GmAP2-7*, were 12.8-14.5, 11.2-11.9, 10.3-11.5, 11.7-14.3, 12.2-14.1, 12.1-;12.7, and 10.9-11.2, respectively, which were all less than that of the wild type control (16.2) (**Figure 4**). Meanwhile, we also observed that these transgenic lines flowered early than the wild type control (**Supplementary Figure S5**). These results indicated that over-expression of these seven *GmAP2* genes led to the early flowering, and these GmAP2 genes were involved in the regulation of flowering time.

**Figure 5:**
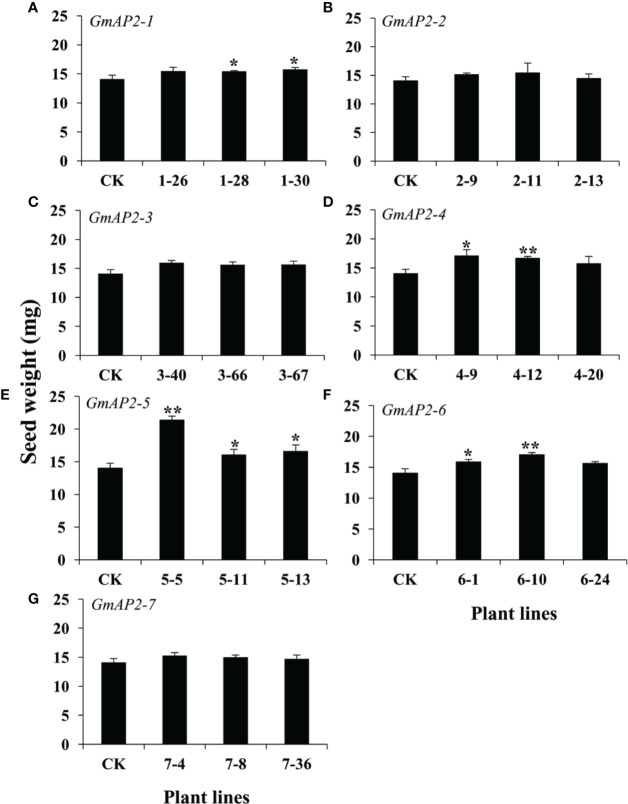
Seed weight of the transgenic lines over-expressing individual *GmAP2* genes in *Arabidopsis*. Seeds were harvested from 10 plants for each line, and seed weight per 1,000 dried mature seeds was measured. CK indicates the wild type *Arabidopsis*. **(A–G)** The over-expression lines for *GmAP2-1* **(A)**, *GmAP2-2* **(B)**, *GmAP2-3* **(C)**, *GmAP2-4* **(D)**, *GmAP2-5* **(E)**, *GmAP2-6* **(F)**, and *GmAP2-7* **(G)**. Data are presented as mean ± SD, Student’s t test (n = 3, *P < 0.05, **P < 0.01).

Besides the regulation of flowering time, *AP2* and *AP2* orthologs are also involved in seed development (Jofuku et al., 1994b; Maes et al., 2001; Jofuku et al., 2005; Ohto et al., 2005; Ohto et al., 2009; Li et al., 2013; Lei et al., 2019). Therefore, we also detected the seed phenotype of these transgenic lines, and found that the thousand seed weight of the *GmAP2-1*, *GmAP2-4*, *GmAP2-5*, and *GmAP2-6* over-expression (OE) lines were 10%, 18%-21%, 14%-52%, and 11%-21% greater, respectively, than the wild type (**Figures 5A, D–F**). But the seed weight of the *GmAP2-2*, *GmAP2-3*, and *GmAP2-7* OE lines showed no significant difference from the wild type (**Figures 5B, C, G****)**.

**Figure 6:**
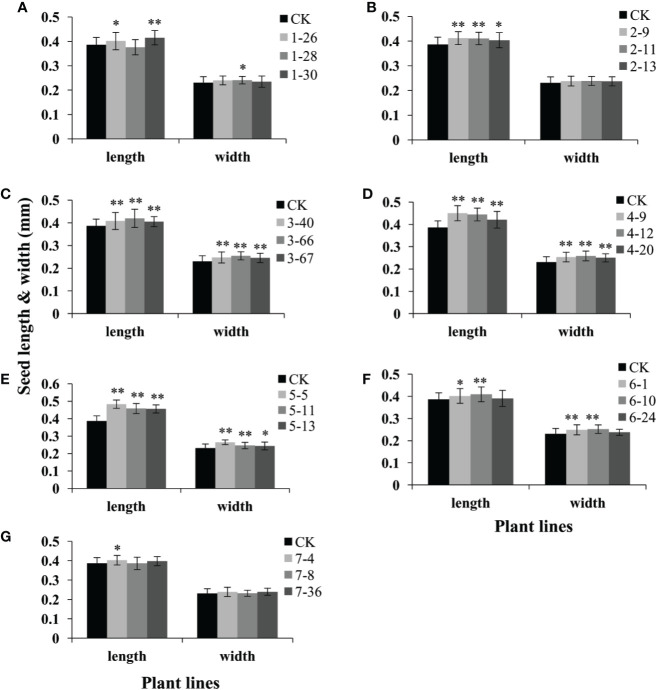
Seed length and width of the transgenic lines over-expressing individual *GmAP2* genes in *Arabidopsis*. Seeds were harvested from 10 plants for each line, and seed length and width of 40 seeds for each line were measured. CK indicates the wild type *Arabidopsis*. **(A–G)** The over-expression lines for *GmAP2-1* **(A)**, *GmAP2-2* **(B)**, *GmAP2-3* **(C)**, *GmAP2-4* **(D)**, *GmAP2-5* **(E)**, *GmAP2-6* **(F)**, and *GmAP2-7* **(G)**. Data are presented as mean ± SD, Student’s t test (n = 40, *P < 0.05, **P < 0.01).

We further found that seed length of the transgenic lines over-expressing these seven genes (*GmAP2-1* to *GmAP2-7*) were 3.9%-7.5%, 4.5%-6.6%, 4.9%-8.6%, 8.9%-16.5%, 18.0%-25.1%, 4.0%-6.0%, and 4.1% longer than the wild type (**Figure 6**). In particular, seed length of the *GmAP2-5* OE lines was 18.0%-25.1% longer than the wild type (**Figure 6E**). The seed width of the OE lines of five genes (*GmAP2-1*, *GmAP2-3*, *GmAP2-4*, *GmAP2-5*, and *GmAP2-6*) were 4.4%, 6.3%-10.3%, 8.4%-12.1%, 5.3%-14.6%, and 7.9%-9.3% longer than the wild type, whereas the OE lines for *GmAP2-2* and *GmAP2-7* genes showed no difference with the wild type in term of seed width (**Figure 6**).

In addition, the seed area of the OE lines of these seven genes were increased by 10.8-12.9%, 3.4%-6.5%, 7.6%-12.7%, 9.3%-13.2%, 6.0%-23.5%, 6.0%-19.0%, and 3.9%-9.5% compared with the wild type (**Supplementary Figure S6**). These results together implied that *GmAP2-1*, *GmAP2-4*, *GmAP2-5*, and *GmAP2-6* functioned in regulating seed weight by affecting seed length, width and area.

**Figure 7:**
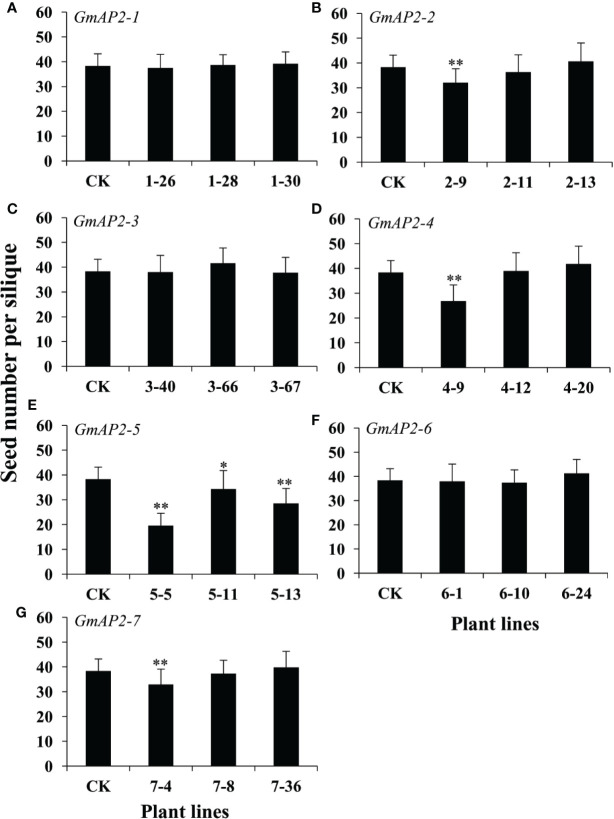
Seed number in each silique of the transgenic lines over-expressing individual *GmAP2* genes in *Arabidopsis*. Thirty-six yellow siliques were collected from 10 plants each line, and seed number per silique was counted. CK indicates the wild type *Arabidopsis*. **(A–G)** The over-expression lines for *GmAP2-1* **(A)**, *GmAP2-2* **(B)**, *GmAP2-3* **(C)**, *GmAP2-4* **(D)**, *GmAP2-5* **(E)**, *GmAP2-6* **(F)**, and *GmAP2-7* **(G)**. Data are presented as mean ± SD, Student’s t test (n = 36, *P < 0.05, **P < 0.01).

It is well known that seed number in each silique is important for seed yield. Therefore, we further calculated seed number per silique of the transgenic lines, and found that seed number per silique in the transgenic lines did not change for the six genes, but that for *GmAP2-5* was obviously reduced compared with the wild type (**Figure 7**). These results showed that these *AP2* genes did not affect seed number per silique except *GmAP2-5*, indicating that these *AP2* genes did not affect plant fertility except *GmAP2-5*. Taken together, three *AP2* genes, *GmAP2-1*, *GmAP2-4*, and *GmAP2-6* control seed yield by affecting seed weight and size in *Arabidopsis*.

### Complementation Assays of *GmAP2* Genes in the *Arabidopsis ap2-6* Mutant

Previous studies showed that the *Arabidopsis ap2-6* mutant flowered earlier than the wild type (Yant et al., 2010), and seed weight and seed size of the *ap2* mutant were increased compared with the wild type (Jofuku et al., 2005; Ohto et al., 2005), with reduced fertility in the strong mutant alleles of *AP2* (*ap2-6*) (Ohto et al., 2005). To determine whether these seven *GmAP2* genes can restore the defective phenotypes (flowering time, seed weight and seed number per silique) of the *ap2-6* mutant, they were individually over-expressed in the *ap2-6* mutant. Our data showed that the flower structure of all the transgenic lines in the *ap2-6* mutant background was similar to the wild type (**Supplementary Figure S7**), indicating that these seven *GmAP2* was able to rescue the defective phenotype of *ap2-6* mutant.

**Figure 8:**
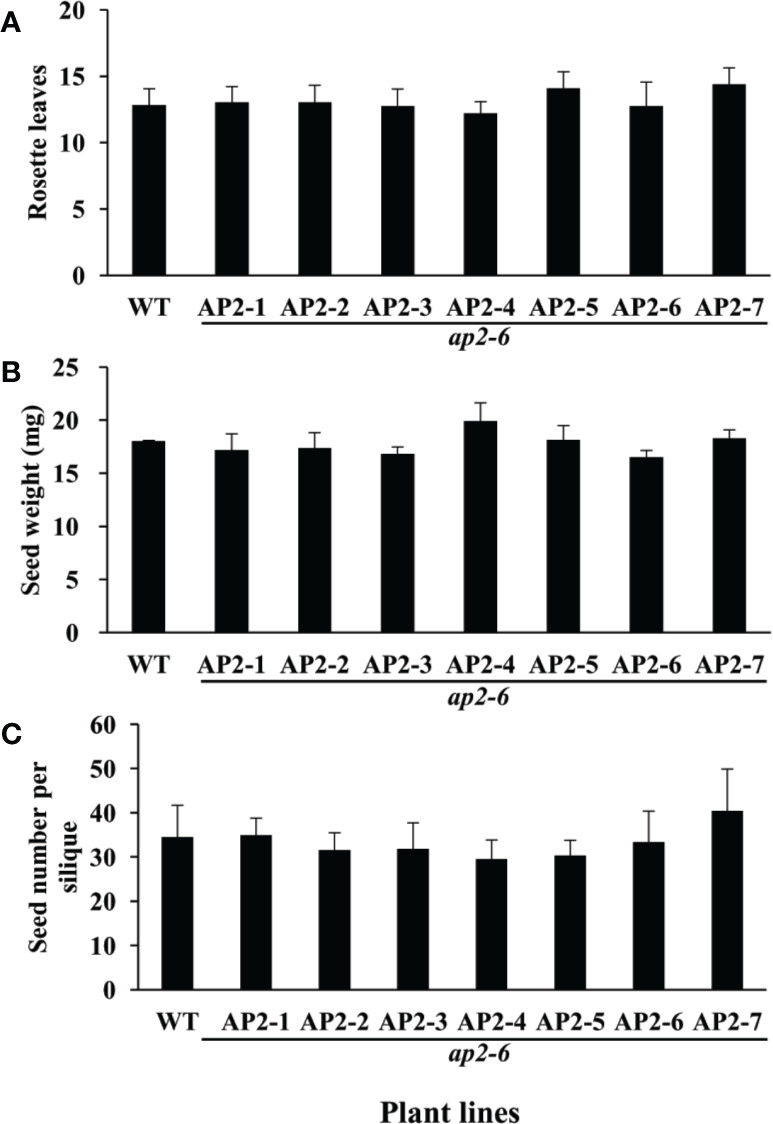
Rosette leave number, seed weight, and seed number in each silique of the transgenic lines over-expressing individual *GmAP2* genes in the *ap2-6* mutant. **(A)** The number of rosette leaves was calculated at the beginning of bolting to evaluate the flowering time of each transgenic line. Twenty plants for each line were selected to count the number of the rosette leaves. Data are presented as mean ± SD, Student’s t test (n = 20, *P < 0.05, **P < 0.01). **(B)** Seeds were harvested from 10 plants for each line, and seed weight per 1,000 dried mature seeds was measured. Data are presented as mean ± SD, Student’s t test (n = 3, *P < 0.05, **P < 0.01). **(C)** Twenty yellow siliques were collected from 10 plants each line, and seed number per silique was counted. WT indicates the wild type *Arabidopsis*. Data are presented as mean ± SD, Student’s t test (n = 20).

The number of rosette leaves in the over-expression lines of the seven *GmAP2* genes in the *ap2-6* mutant showed no significant difference from the wild type (**Figure 8A**). We further found that seed weight of the individual over-expression lines of these *GmAP2* genes in the *ap2-6* mutant were same as the wild type (**Figure 8B**), but they were far lower than those of the *ap2-6* mutant (Jofuku et al., 2005; Ohto et al., 2005). Their seed number per silique was also the same as the wild type (**Figure 8C**), but was higher than 23 ± 8.1 seeds of the *ap2-6* mutant (Ohto et al., 2005). Taken together, these results indicated that these seven *GmAP2* genes could restore the defective phenotypes of early flowering, large seed and less seed number of the *Arabidopsis ap2-6* mutant.

## Discussion

Soybean is the world’s largest single source of vegetable protein and oil, accounting for more than 50% of the world edible oil (Soystats 2013, http://www.soystats.com). Same as for the other major crops, grain yield is always the most important trait in soybean breeding and cultivation, and improvement of seed yield is one of the major objectives in soybean breeding. Previous studies showed that both the time of flowering and date of seed maturity underlie seed yield (Yuan et al., 2002), and the ovule and seed development control final seed weight (Jofuku et al., 1994b; Jofuku et al., 2005; Ohto et al., 2005; Ohto et al., 2009). The *APETALA2* (*AP2*) gene in *Arabidopsis*, as a member of AP2/ERF superfamily, regulates floral development (Jofuku et al., 1994a; Yant et al., 2010), and ovule and seed development, and subsequently mediates seed size and seed weight (Jofuku et al., 1994b; Jofuku et al., 2005; Ohto et al., 2005; Ohto et al., 2009).

In this study, we identified all 380 AP2/ERF superfamily members in *G. max*, which is more than two folds of the numbers in *Arabidopsis*. Meanwhile, the numbers of the ERF and the AP2 family in *G. max* are more than doubled than in *Arabidopsis*, respectively (**Figure 1**, **Supplementary Figures S1** and **S2**). These results indicated that the AP2/ERF superfamily members appeared to increase proportionally, compared with *Arabidopsis*. We also found that the euAP2 lineage in *G. max* (**Figure 1**), and the number of miR172 in *G. max* that can target the euAP2 lineage (Wang et al., 2014), are more than doubled than the number of *Arabidopsis*. Compared with *Arabidopsis*, the numbers of miR172 family and their target genes (the genes of the euAP2 lineage) in *G. max* also appeared to increase proportionally. The same phenomenon was found for other large transcription factor families. Previous studies showed that the members of WRKY and R2R3-MYB family in *G. max*, are about two folds of the number in *Arabidopsis* (Chen et al., 2006; Du et al., 2012; Phukan et al., 2016; Yu et al., 2016b). The gene numbers of the same family were increased, which suggested that some genes developed new function during evolution.

Recent studies showed that miR172c in soybean targets the *AP2* transcription factor *NNC1* to activate the expression of *ENOD40* gene (Wang et al., 2014), and miR172 in *Arabidopsis* targets all the six members of the euAP2 lineage (*AP2*, *TOE1*, *TOE2*, *TOE3*, *SMZ* and *SNZ*) to regulate flowering time (Yant et al., 2010). Another study showed that typical *AP2* genes firstly appear in gymnosperms and diverged in angiosperms, following expansion of group members and functional differentiation (Wang et al., 2016). The gene numbers of the same family were increased, which also suggested that more genes functioned redundantly. miRNAs often showed conserved function in different species, and miR172 in soybean and their target genes had more members than those of *Arabidopsis*, indicating that more euAP2 genes functioned redundantly in soybean. Our study showed that at least four *AP2* family members in soybean were involved in seed weight and seed size, in contrast, only single *AP2* members in *Arabidopsis* regulates seed weight and seed size (Jofuku et al., 2005; Ohto et al., 2005). Compare with *Arabidopsis*, more AP2 family members in soybean played redundant role in regulating seed weight and seed size. Based on these evidences, it is believed that AP2/ERF superfamily members in *G. max* possibly are redundant with more diverse functions compared with *Arabidopsis*.

These seven *AP2* orthologs from soybean have a partially conserved role in floral organ identity with *AtAP2* as in other plant species. Previous studies showed that the *AP2* gene is expressed in all four floral organs including sepal, petal, stamen and carpel, and affects floral organ identity specification (Drews et al., 1991; Jofuku et al., 1994a). It was observed that the homeotic transformation of sepals into carpels in *ap2-5*, *ap2-6*, and *ap2-7*; of petals to stamens in *ap2-5*; of petals to carpels in *ap2-6* (Kunst et al., 1989). Our study showed that these 7 *GmAP2* genes were expressed in flowers (**Figure 3**) and were able to rescue the defective phenotype of *ap2-6* mutant, but their over-expression did not produce excess floral organs (**Supplementary Figure S7**), indicating that they retained partially conserved function in floral organ identity. Other studies showed that many *AP2* orthologs in other species were also expressed in floral organs; and the mutation of *AP2* orthologs led to the defective floral organ, and their over-expression produce excess floral organs; these *AP2* orthologs generally rescue the defective phenotype of floral organ identity in the *Arabidopsis ap2* mutant (Keck et al., 2003; Nilsson et al., 2007; Liu et al., 2012; Luo et al., 2012; Yan et al., 2012; Zhang et al., 2018). Therefore, the *AP2* genes of different plant species, including these 7 *GmAP2* genes, may share conserved roles in floral organ identity.

The *Arabidopsis AP2* and *AP2* orthologs in other species were reported to repress the flowering time, and their mutants led to earlier flowering, and their over-expression lines flower later compared with the wild type. For example, the *ap2* mutants and other members of the euAP2 lineage in *Arabidopsis* flowered earlier than the wild type (Yant et al., 2010). The flowering time was delayed by 8-9 days in plants over-expressed *LaAP2L1* from *Larix* compared with the vector control plants (Li et al., 2013). Over-expression of the *AfAP2-2* gene from *Aechmea fasciata* delays flowering in *Arabidopsis* (Lei et al., 2019). The transgenic *PaAP2L2* plants flowered later than control plants (Nilsson et al., 2007). Our data indicated that these seven genes were able to rescue the earlier flowering phenotype in *ap2-6* mutant. However, in contrast to the late flowering phenotype in the over-expression plants of the *AP2* orthologs (*LaAP2L1*, *AfAP2-2*, and *PaAP2L2*), the over-expression of these seven *GmAP2* genes led to early flowering. Although these seven *GmAP2* genes were able to rescue the early flowering time phenotype of *ap2-6* mutants, which is different from the other *AP2* genes, indicating that they had no similar role in regulating flowering time with other *AP2* genes. The molecular mechanism of functional differentiation between *GmAP2* and other *AP2* orthologs (*LaAP2L1*, *AfAP2-2* and *PaAP2L2*) needs further investigation.

It is well known that *AP2* in *Arabidopsis* and *AP2* orthologs in other species play key roles in seed development. It was indicated that *AP2* and *AP2* orthologs in *Petunia hybrid*, *Aechmea fasciata* and *Brassica napus* negatively regulated seed weight and seed size. For example, *AP2* negatively regulates seed weight and seed size in *Arabidopsis* (Jofuku et al., 2005; Ohto et al., 2005). In addition, *PhAp2A* is capable of restoring the seed defective phenotype of the *Arabidopsis ap2-1* mutant (Maes et al., 2001); and the seed size of *Arabidopsis* over-expressing *AfAP2-2* gene from *Aechmea fasciata* was reduce (Lei et al., 2019). In the RNAi lines for the *BnAP2* gene, seeds showed defects in shape, structure and development and larger seed size (Yan et al., 2012). However, another study reported that over-expression of *LaAP2L1* from *Larix* led to larger organ size and over 200% greater seed yield in *Arabidopsis*, due to enhanced cell proliferation and prolonged growth duration (Li et al., 2013), indicating that the *AP2* ortholog from *Larix* positively regulated seed weight and seed size. Our data suggested that *GmAP2-1*, *GmAP2-4*, *GmAP2-5*, and *GmAP2-6* positively regulated seed weight and seed size, which is the same as *LaAP2L1*.

In addition, the seed weight of individual over-expression lines of these *GmAP2* genes in *ap2-6* mutant was not obviously different from that of the wild type (**Figure 8B**), and was significantly decreased compared with that of the *ap2-6* mutant, suggesting that these *GmAP2* genes have a partial conserved negative role in regulating seed weight and seed size. In comparison, the function of *GmAP2* in regulating seed yield and size in *Arabidopsis* was positive by over-expression and negative in the complimentary assay, which is different from AP2 orthologs of other plant species.

Our studies implied that *GmAP2-1*, *GmAP2-4*, *GmAP2-5*, and *GmAP2-6* function in regulating seed weight by affecting seed length, width and area, but *GmAP2-1*, *GmAP2-4* and *GmAP2-6* did not affect seed number per silique (**Figure 7**). Taken together, *GmAP2-1*, *GmAP2-4*, and *GmAP2-6* genes control seed yield by affecting seed weight and seed size, and they could be utilized as potential target genes for seed yield breeding in soybean.

## Data Availability Statement

The datasets presented in this study can be found in online repositories. The names of the repository/repositories and accession number(s) can be found in the article/supplementary material.

## Author Contributions

WJ and YP conceived the project, designed the experiments. WJ, XZ, and XS performed the experiments and analyzed the data. WJ and XZ prepared the manuscript. JY and WJ performed phylogenetic analysis. YP revised the manuscript and supervised the project.

## Funding

This work was supported by the National Key Research and Development Plan (2016YFD0101005). This work was also supported by the National Natural Science Foundation of China (31870281).

## Conflict of Interest

The authors declare that the research was conducted in the absence of any commercial or financial relationships that could be construed as a potential conflict of interest.
