# Supplementary material for: Genome-Wide Identification and Characterization of APETALA2/Ethylene-Responsive Element Binding Factor Superfamily Genes in Soybean Seed Development
Source: Front Plant Sci. 2020 Sep 4;11:566647. doi: 10.3389/fpls.2020.566647 (PMC7498640; doi:10.3389/fpls.2020.566647)
Supplement: Supplementary file 1 [file DataSheet_1.pdf]

Supporting Information

Supplementary Table S1. Sequence identities (%) of nine AP2 proteins of *G. max*.

Gene Name	AtAP2	GmAP2-1	GmAP2-2	GmAP2-3	GmAP2-4	GmAP2-5	GmAP2-6	GmAP2-7	GmNNC1
AtAP2	100	60.5	77.01	79.33	57.19	54.44	53.85	52.33	61.76
GmAP2-1		100	73.33	73.3	58.23	53.01	52.72	54.17	62.26
GmAP2-2			100	73.13	55.52	55.03	53.85	52.91	77.93
GmAP2-3				100	90.13	52.2	52.63	50.87	74.8
GmAP2-4					100	47.06	53.85	52.6	74.4
GmAP2-5						100	75.11	69.77	54.82
GmAP2-6							100	70.06	53.94
GmAP2-7								100	52.07
GmNNC1									100

Supplementary Table S2. The primer sequences used in the present study

Purpose	Primer name	Primer sequences (5’-3’)
For gene cloning	GmAP2-1F	CACCATGTGGGATCTGAATGACTC
	GmAP2-1R	TCATGAGGGTCTCATGAGAGA
	GmAP2-2F	CACCATGTGGGATCTCAACGACTCA
	GmAP2-2R	TCATGAGGGTCTCATGAGACTG
	GmAP2-3F	CACCATGTTGGATCTTAATCTGAATG
	GmAP2-3R	TCAGAACTTGTGTGGTGGGCTAC
	GmAP2-4F	CACCATGTTGGATCTTAATCTGACTG
	GmAP2-4R	CTAGGACGGTGGCTGCGGGGACTT
	GmAP2-5F	CACCATGAGTAACTGGTTGGGGTTCT
	GmAP2-5R	TCATTCATTCCACAAAGCAAACA
	GmAP2-6F	CACCATGGCTGGTGCCACGAATTGG
	GmAP2-6R	TCAGTAGGACTGGTGAGGCCATA
	GmAP2-7F	CACCATGAAGAGGTCTCCAGCATCTTC
	GmAP2-7R	TCATAGATCTAGAGCATAGTCAC
For qRT-PCR analysis	qGmAP2-1F	AGGTGGAAGCAGAACATTGGATA
	qGmAP2-1R	ACTGTCATCATGAGCTACAGAG
	qGmAP2-2F	CTTCCTTCGATGGAGATGATGAC
	qGmAP2-2R	CTGTCCATGGACTCTTCTTCATT
	qGmAP2-3F	CCCGCTGGATCATCAAAACGGT
	qGmAP2-3R	CGGTCCTTCTGTAGAAAGTGA
	qGmAP2-4F	AGTCACCTTCTACCGAAGAACC
	qGmAP2-4R	TAGATCCTCCTCATAATCAACG
	qGmAP2-5F	GGCACCAAGCTTTTGCACCAA
	qGmAP2-5R	CAAGGAAGAAGGATTTGTGAGAT
	qGmAP2-6F	GGACCAACTCGGGGTCCGA
	qGmAP2-6R	GTCAAAGCTGATCCTTTACAATTA
	qGmAP2-7F	AGAAGGGTCGACAAGTTTATTTG
	qGmAP2-7R	TTCCGCATACTCTTCCAATTCGT
	qPP2A-F	TATCGGATGACGATTCTTCGTGCAG
	qPP2A-R	GCTTGGTCGACTATCGGAATGAGAG
	qSUB3-F	GTGTAATGTTGGATGTGTTCCC
	qSUB3-R	ACACAATTGAGTTCAACACAAACCG

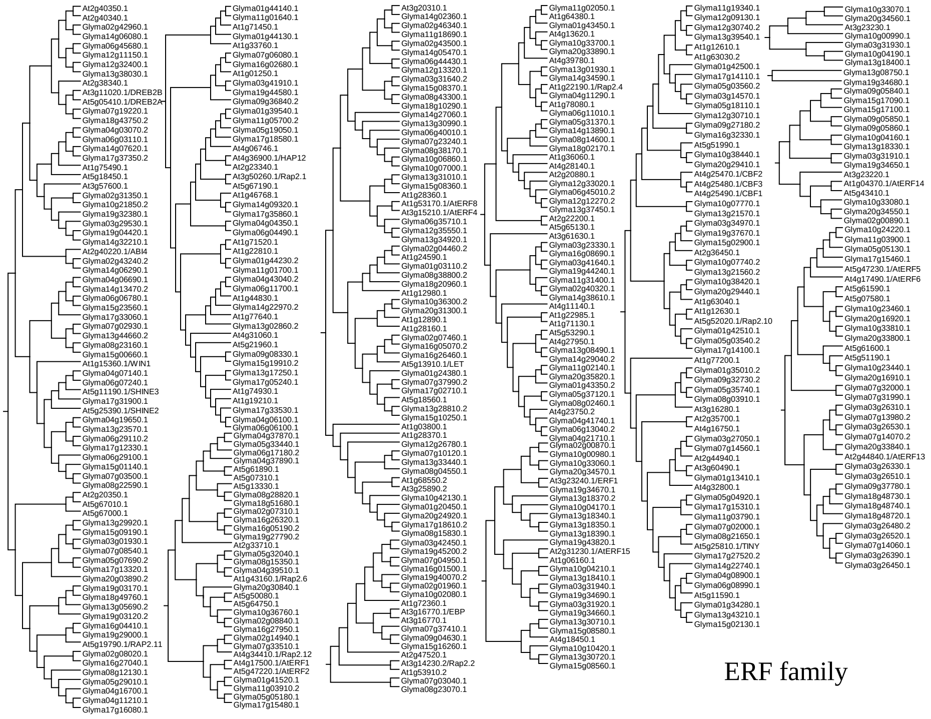

Figure. S1. Phylogenetic analysis of the ERF family members in soybean and *Arabidopsis*. The neighbor-joining (NJ) method was applied to construct trees using MEGA X software. Bootstrapping with 500 replications was performed.

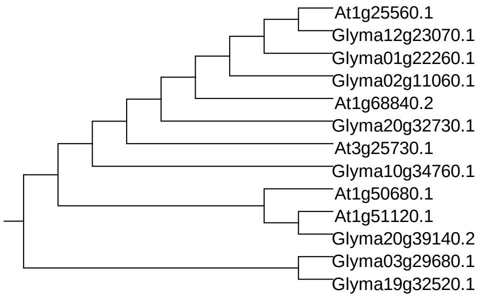

RAV family

Figure. S2. Phylogenetic analysis of the RAV family members in soybean and *Arabidopsis*. The neighbor-joining (NJ) method was applied to construct trees using MEGA X software. Bootstrapping with 500 replications was performed.

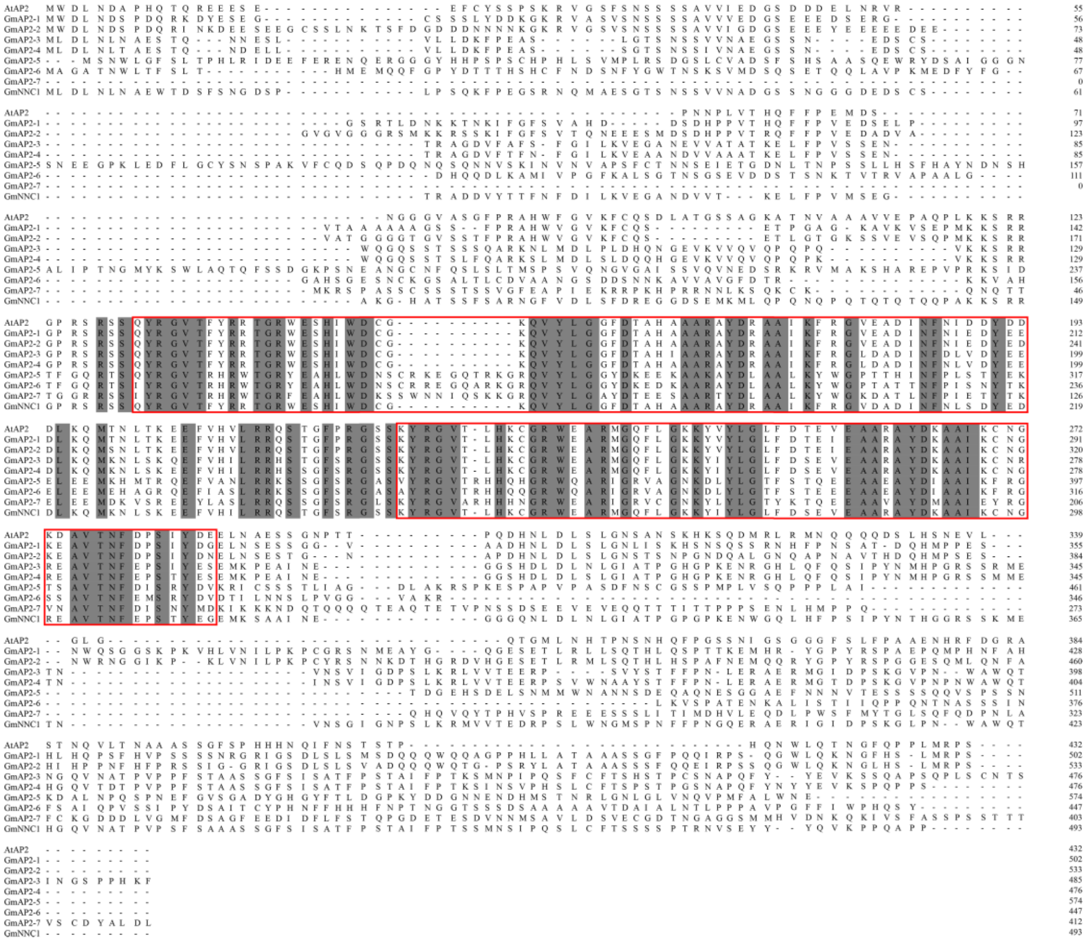

Figure. S3. Sequence alignment of the candidate AP2 proteins in soybean with those from *Arabidopsis*. The alignment was performed by using software DNAMAN. The two AP2 domains were boxed with red color. All the sequences for soybean used in the figure are retrieved from the Soybean Database (http://soykb.org/) and AtAP2 are retrieved from the Arabidopsis Database (https://www.arabidopsis.org/). The accession number are as followed (shown in parenthesis): *Arabidopsis thaliana*: AtAP2 (At4g36920); *Glycine max*: GmAP2-1 (Glyma01g39520.3), GmAP2-2 (Glyma05g18041.1), GmAP2-3 (Glyma15g04930.1), GmAP2-4 (Glyma13g40470.1), GmAP2-5 (Gm16g00950.2), GmAP2-6 (Glyma08g38190.2), GmAP2-7 (Glyma15g34770.1) and GmNNC1 (Glyma12g07800.1).

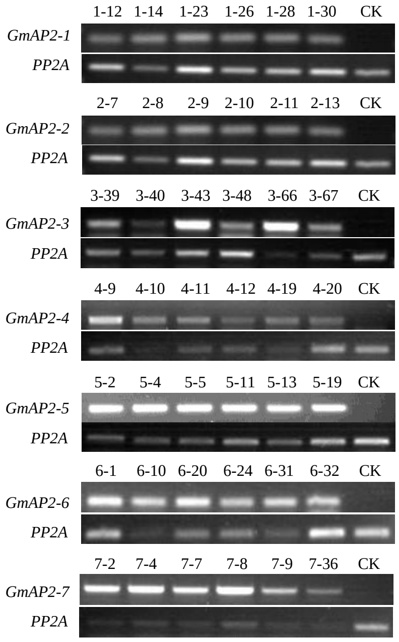

Figure. S4. Relative transcript levels of the candidate *GmAP2* genes in the transgenic *Arabidopsis* lines detected by RT-PCR. CK represents *Arabidopsis* wild type, the other numbers represent different transgenic lines in T_1 generation (*GmAP2-1*, *GmAP2-2*, *GmAP2-3*, *GmAP2-4*, *GmAP2-5*, *GmAP2-6*, *GmAP2-7*). The *PP2A* gene (At1g13320) was used as the reference gene.

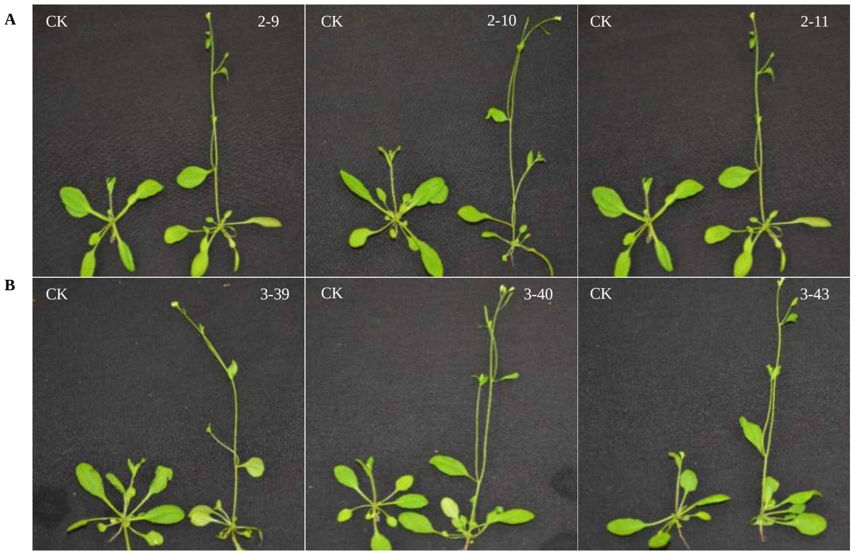

Figure. S5. Representative early flowering of the *GmAP2* over-expression lines in *Arabidopsis*. All plants were grown in the same tray to ensure the same growth condition (16-h light/8-h dark) at 22 ℃. The 30-day-old plants were photographed. (A) The *GmAP2-2* over-expression lines (2-9, 2-10 and 2-11) in *Arabidopsis*. (B) The *GmAP2-3* over-expression lines (3-39, 3-40 and 3-43) in *Arabidopsis*. CK, the wild type *Arabidopsis* as control.

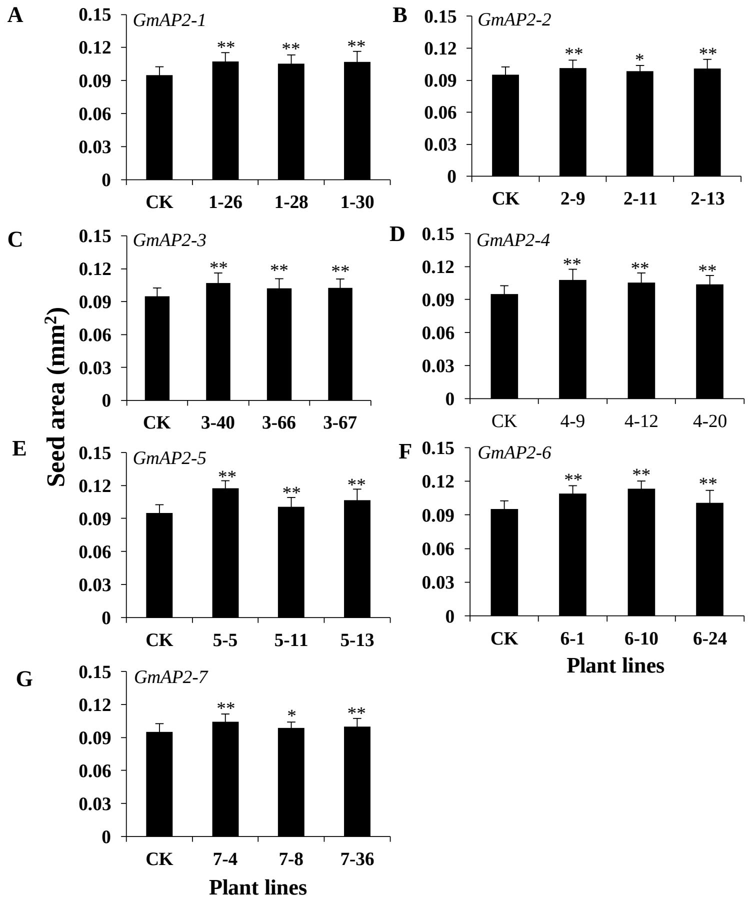

Figure. S6. Seed area of the transgenic lines over-expressing seven individual *GmAP2* genes in the *Arabidopsis* wild type. Seeds were harvested from 10 plants each line, and 40 seeds each line were photographed. ImageJ software was used to calculate the seed area. CK indicates the wild type *Arabidopsis*. Data are presented as mean ±SD, Student's t test (n=40, *P <0.05, **P <0.01). (A-G) The over-expression lines for *GmAP2-1* (A), *GmAP2-2* (B), *GmAP2-3* (C), *GmAP2-4* (D), *GmAP2-5* (E), *GmAP2-6* (F), *GmAP2-7* (G).

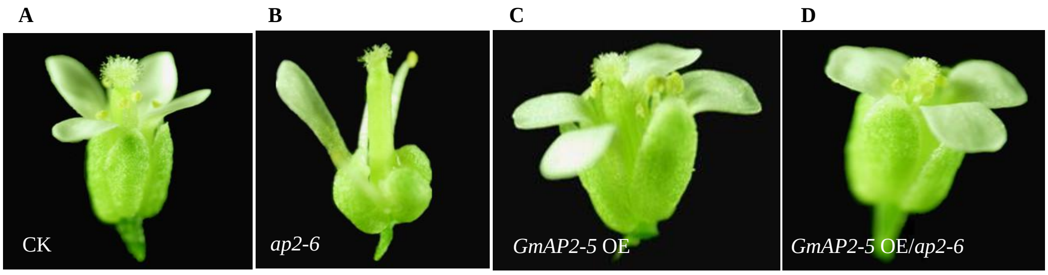

Figure. S7. Representative flower structure of the *GmAP2* over-expression lines in *Arabidopsis*. All plants were grown in the same tray to ensure the same growth condition (16-h light/8-h dark) at 22 ℃. The flowers of the 42-day-old plants were photographed. (A) CK, the wild type *Arabidopsis* as control. (B) The *ap-6* mutant line. (C) The transgenic lines over-expressing *GmAP2-5* in the wild type. (D) The transgenic lines over-expressing *GmAP2-5* in the *ap-6* mutant background.
